# Supplementary material for: Lipid droplet‐dependent fatty acid metabolism controls the immune suppressive phenotype of tumor‐associated macrophages
Source: EMBO Mol Med. 2019 Oct 10;11(11):e10698. doi: 10.15252/emmm.201910698 (PMC6835560; doi:10.15252/emmm.201910698)
Supplement: Supplementary file 1 — Appendix [file EMMM-11-e10698-s001.pdf]

Supplementary Materials

Table of contents:

Figure S1. Oleate-polarized Gr1$^-$CD11b$^+$ myeloid cells promote tumor growth.

Figure S2. PGE2 controls the inhibitory function but not the phenotype of Gr1-CD11b+ myeloid cells.

Figure S3. Diminished polarization of CD206$^+$ myeloid cells by SCD1 inhibitor can be neutralized by exogenous oleate.

Figure S4. Size measurement of the encapsulated liposome with several inhibitors.

Figure S5. Both, bone marrow hematopoietic stem cells and monocytes can polarize into CD206+ suppressive myeloid cells.

Figure S6. Lipid droplet analysis in TAMs of gastro-esophageal cancer patients.

Table S1. Significantly distinct pathways between control and oleate group based on enrichment score and p-value from microarray.

Table S2. Supplementary Material and Methods: Antibody dilutions.

Table S3. Supplementary Material and Methods: Exact p-values and statistical tests.

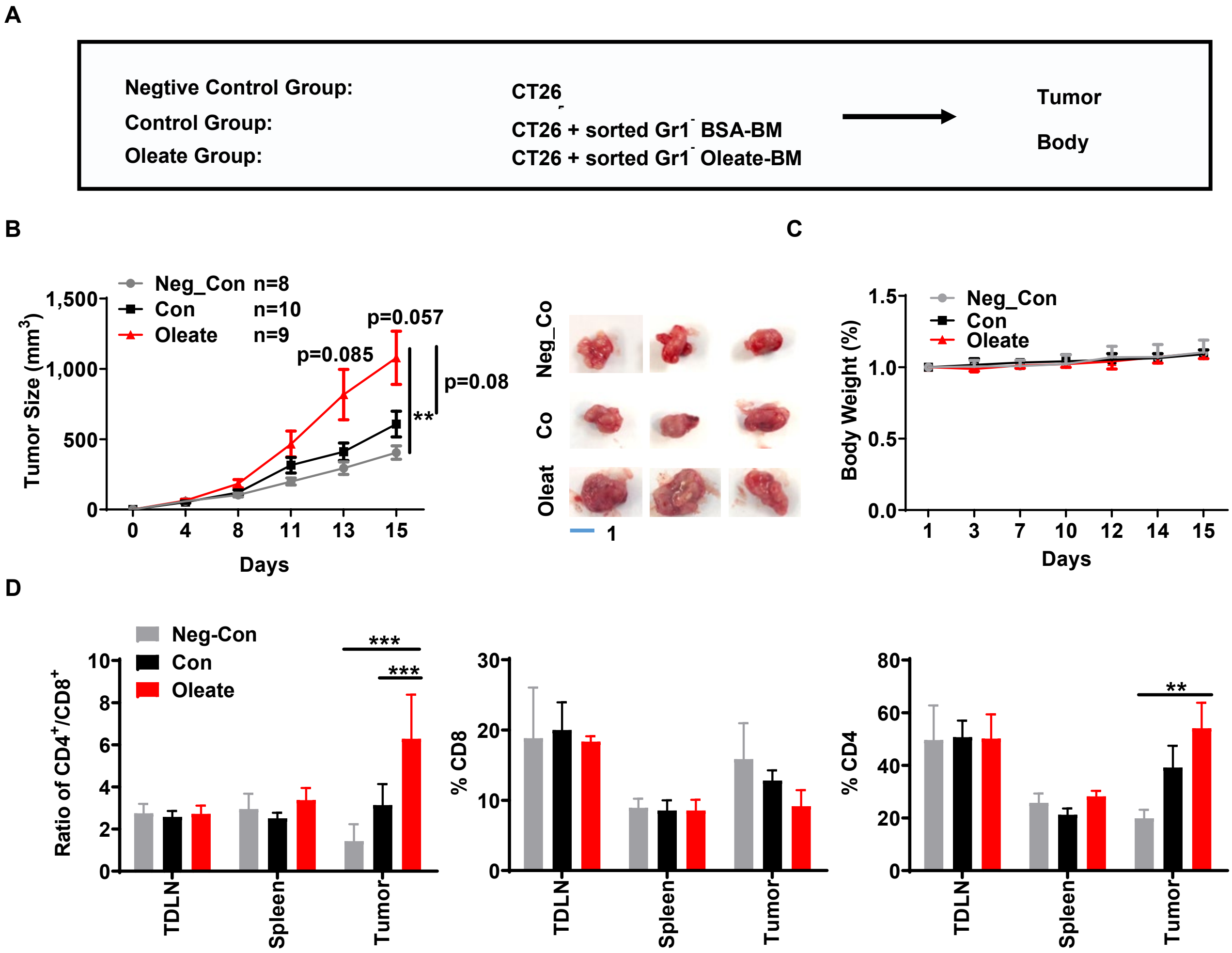

Fig. S1. Oleate-polarized Gr1$^-$CD11b$^+$ myeloid cells promote tumor growth.

(A) The experimental procedure to test the pro-tumoral function of Gr1$^-$CD11b$^+$ myeloid cells is shown. 5×10^5 CT26 colon carcinoma cells were mixed with myeloid cells in the ratio of 1:1 and inoculated into Balb/c mice. (B) Tumor growth as well as (C) body weight were monitored every 2-4 days. Tumor bearing mice were sacrificed on day 15. (D) Tumors were isolated and digested for immune cell analysis via flow cytometry. Shown is the mean±SEM from two to four independent experiments. Two-way analysis of variance (ANOVA) with Tukey's post hoc test was performed to compare the tumor growth as well as immune cell analysis. $^*p<0.05$; $^{**}p\leq0.01$

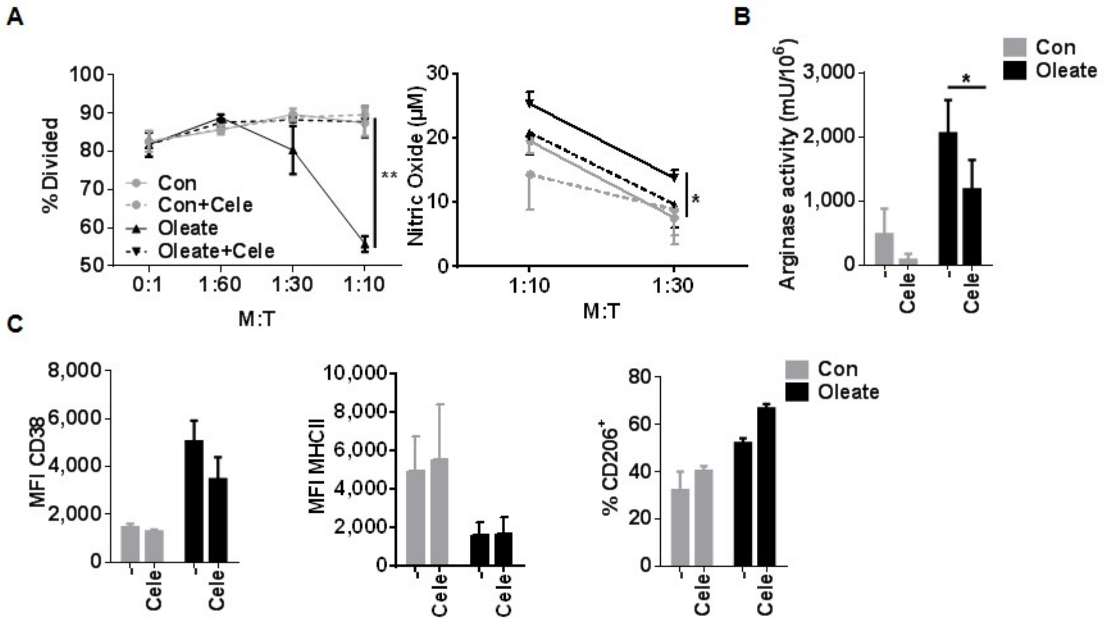

Fig. S2. PGE₂ controls the inhibitory function but not the phenotype of Gr1⁻CD11b⁺ myeloid cells.

Bone marrow cells were isolated and polarized in the presence or absence of 5 μM celecoxib (Cele) in the presence of 40 ng/ml GM-CSF for 6 days. (A) T cell proliferation assay was performed via co-culture with purified $CD4^+$ T cell in the ratios indicated. (B) 1×10^6 myeloid cells after polarization were lysed for arginase activity assay. (C) The expression of CD38, MHCII as well as the proportion of $CD206^+$ myeloid cells was analyzed via flow cytometry. Shown is the mean±SD from two to four independent experiments. Two-way ANOVA with Tukey's post hoc test was performed to compare the T cell proliferation in oleate group ± celecoxib treatment. *p<0.05

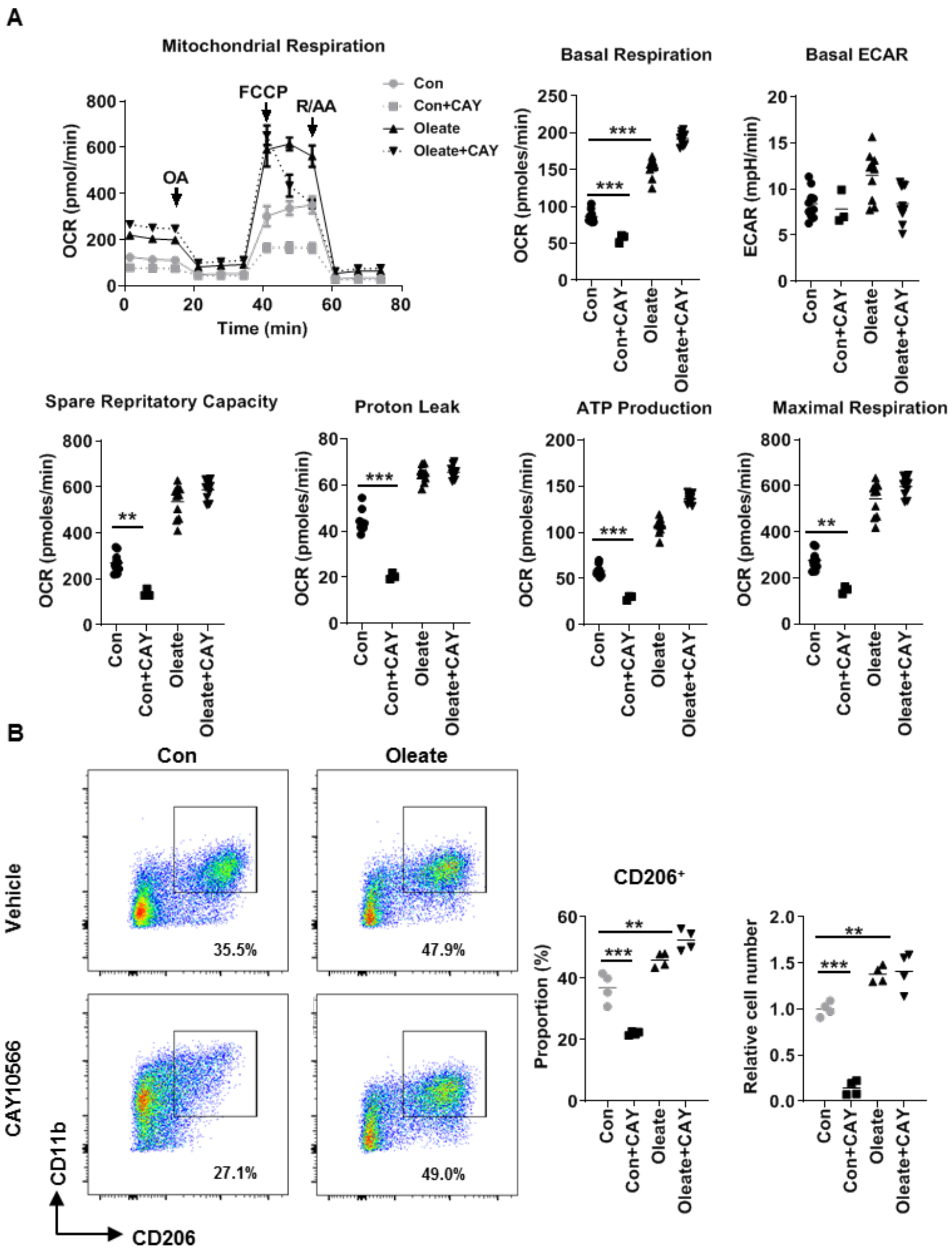

Fig. S3. Diminished polarization of CD206^{+} myeloid cells by SCD1 inhibitor can be neutralized by exogenous oleate.

Bone marrow cells were isolated and polarized in the presence or absence of 10 nM SCD1 inhibitor CAY10566 in the presence of 40 ng/ml GM-CSF for 6 days. (A) The oxygen consumption rate of $1{\times}10^{5}$ purified Gr1^{-} cells was monitored after the addition of oligomycin (OA) (1 µM), the uncoupler carbonyl cyanide-4-(trifluoromethoxy) phenylhydrazone (FCCP, 1 µM), and the electron transport inhibitor rotenone and antimycin A (R/AA) (0.5 µM) at

indicated time points. The value of basal oxygen consumption rate (OCR), basal extracellular acidification rate (ECAR), spare respiratory capacity, proton leak, ATP production and maximal respiration were calculated. (B) The percentage and absolute number of $CD206^+$ suppressive myeloid cells were calculated based on flow cytometry. Shown is the mean±SD from two to four independent experiments. Two-way ANOVA with Tukey's post hoc test was performed to compare the effect of CAY10566 in control and oleate group. $^{**}p \leq 0.01$; $^{***}p \leq 0.001$

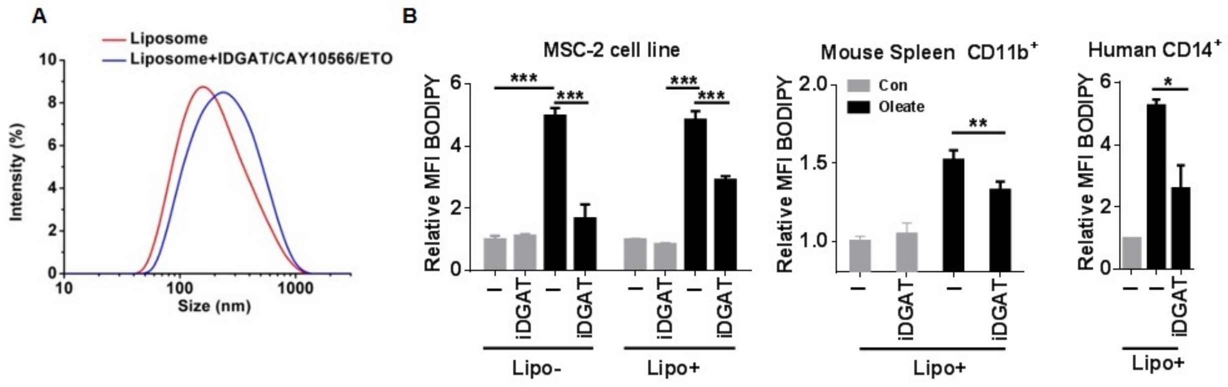

Fig. S4. Size measurement of the encapsulated liposome with several inhibitors.

(A) The sizes of control vehicle and liposomes encapsulated with inhibitors were measured by a laser particle size analyzer at a wavelength of 633 nm with a constant angle of 173 °. The diameter of micelles was calculated from the average of three individual experiments. (B) To validate the efficiency of iDGAT-liposome, MSC-2 cell line, splenocytes or human CD14$^+$ monocytes were treated with oleate. Twenty-four hours later, cells were collected for BODIPY and antibody staining for the indicated markers. Shown is the mean±SEM from two to four independent experiments. One-way ANOVA was performed to compare the effect of iDGAT-encapsulated liposome in MSC-2 cell line, mouse spleen CD11b$^+$ cells and human CD14$^+$ cells. *$p<0.05$; **$p\leq0.01$

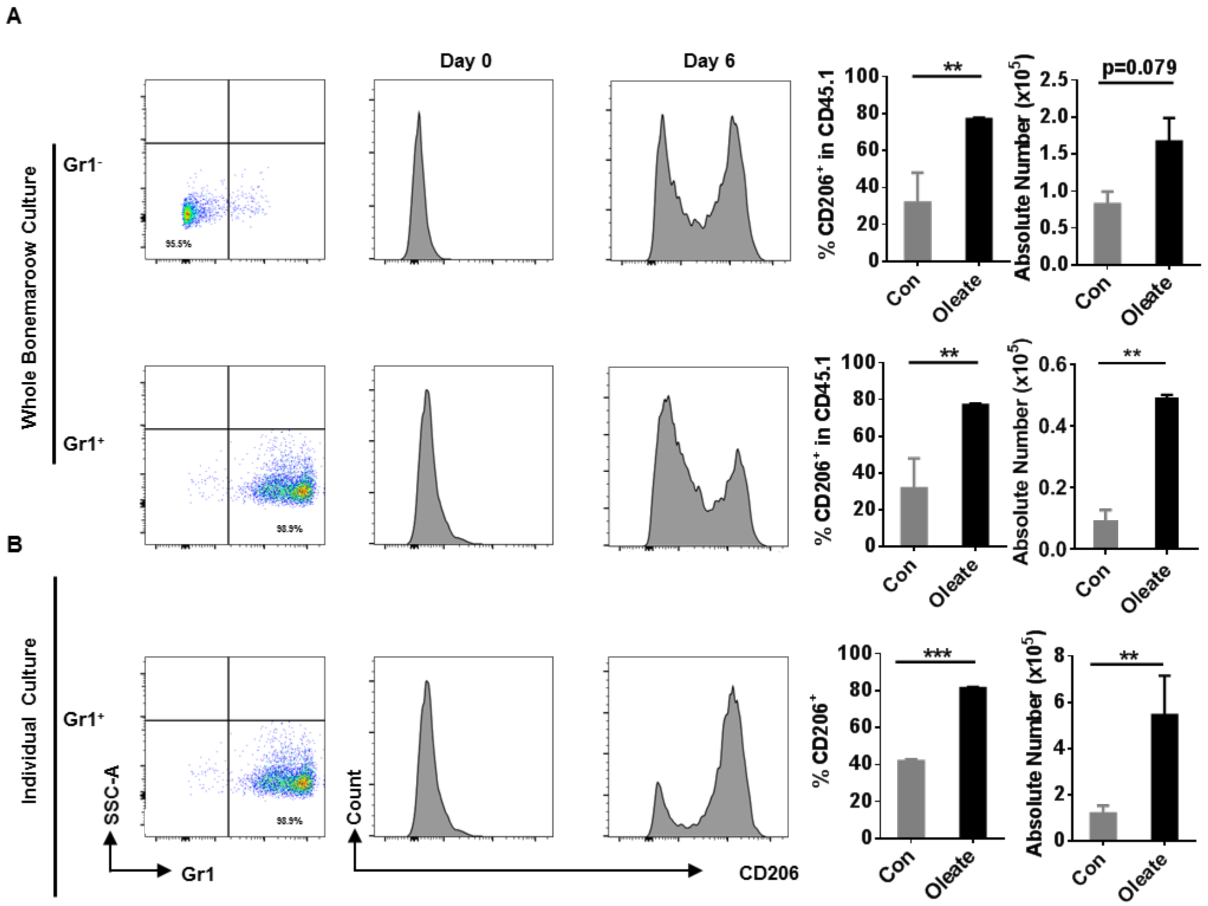

Fig. S5. Both, bone marrow hematopoietic stem cells and monocytes can polarize into CD206$^+$ suppressive myeloid cells.

Gr1$^-$ and Gr1$^+$ cells bone marrow (CD54.1$^+$) were isolated and mixed with CD45.2$^+$ bone marrow cells for polarization assay in the presence of oleate. (A) The frequency of CD45.1$^+$CD206$^+$ myeloid cells was analyzed via flow cytometry on day 6. (B) To avoid the impact of other cells, the same bone marrow purified Gr1$^+$ cells were also individually cultured for 6 days. Shown is the mean±SD from two to four independent experiments. Unpaired Student's two-tailed t tests were performed to compare the percentage of CD206$^+$ cells in different groups. $^{**}p \leq 0.01$; $^{***}p \leq 0.001$

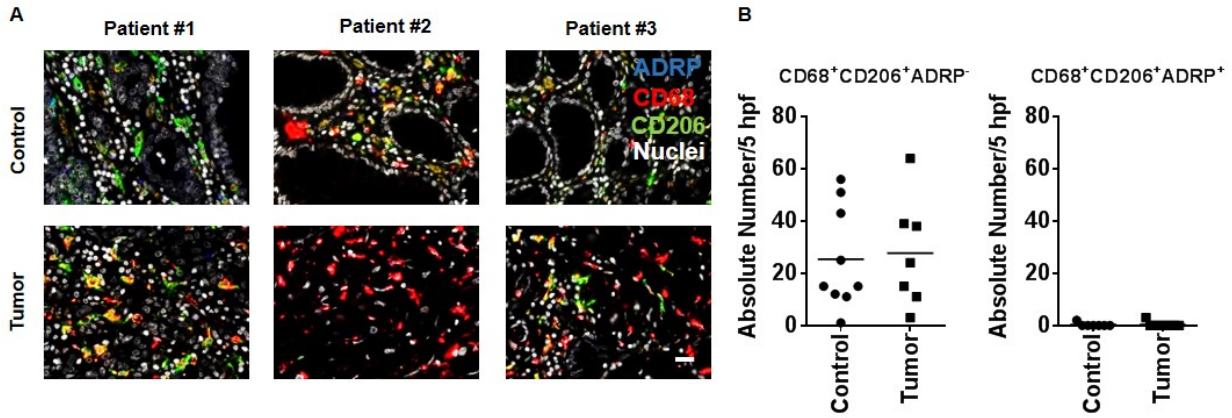

Fig. S6. Lipid droplet analysis in TAMs of gastro-esophageal cancer patients.

Tumor tissue (Tumor) as well as corresponding adjacent tissue (Control) from gastro-esophageal cancer patients served for histology staining. (A) CD68 (red), CD206 (green) and ADRP (blue) staining were performed to identify the lipid droplets in tumor infiltrating myeloid cells. Nuclei are visible in white (scale bar = 20 μm). (B) The absolute number of double/triple positive cells in 5 high-power fields (hpf) was determined.

Biological Process/ Cellular Component	Enrichment Score	Enrichment p-value	% genes in group that are present	# genes in list, in group	GO ID
**antigen processing and presentation of peptide or polysaccharide antigen via MHC class II**	24.3938	2.54622e-011	80	8	2504
**MHC class II protein complex**	23.1209	9.09299e-011	72.7273	8	42613
**response to hypoxia**	10.0506	4.31603e-005	9.13978	17	1666
**response to interferon-γ**	9.74438	5.86234e-005	25	6	34341
**sprouting angiogenesis**	8.03979	0.000322378	18.75	6	2040
**positive regulation of angiogenesis**	6.82014	0.00109157	9.61538	10	45766
**negative regulation of activated T cell proliferation**	5.27041	0.00514148	50	2	46007

Table S1. Significantly distinct pathways between control and oleate group based on enrichment score and *p*-value from microarray.

Antibody	Dilution
Flow cytometry	
Mouse CD4 (clone GK1.5) APC	1:400
Mouse CD8a (clone 53-6.7) FITC	1:400
Mouse CD11b (clone M1/70) APC-Cy7	1:400
Mouse CD11c (clone N418) PerCP-Cy5.5	1:400
Mouse MHCII (clone M5-114.15.2) APC	1:400
Mouse Gr1 (clone RB6-8C6) APC	1:1000
Mouse CD38 (clone HIT2) eFluor 450	1:200
Mouse CD73 (clone TY/11.8) PE-Cy7	1:200
Mouse CD206 (clone MR5D3) Alexa488	1:50
Human CD204 (clone UC23-56) PE	1:50
Human CD206 (clone 19.2) eFluor 450	1:50
Human CD38 (clone HB7) PE-Cy7	1:50
Human CD73 (clone AD2) FITC	1:50
**Immunohistochemistry**	
Mouse CD8a (clone D4W2Z)	1:200
Human ADRP (Rabbit Polyclonal)	1:800
Human CD68 (clone PG-M1)	1:250
Human CD206 (clone 5C11)	1:500
**Western Blot**	
Mouse mTOR (Rabbit Polyclonal)	1:1000
Mouse mTOR (pSer 2448) (Rabbit Polyclonal)	1:1000
Mouse mTOR (pSer 2481) (Rabbit Polyclonal)	1:1000
Mouse anti-b-actin (clone AC-15)	1:2000

Table S2. Supplementary Material and Methods: Antibody dilutions.

Figure	Comparison	P value	Statistic Methods
Fig1.C-CD11c	Con vs Oleate	0,0083	Student t-test
Fig1.C-MHCII	Con vs Oleate	0,0105	Student t-test
Fig1.C-CD206	Con vs Oleate	0,0077	Student t-test
Fig1.C-CD73	Con vs Oleate	0,0148	Student t-test
Fig1.C-CD38	Con vs Oleate	0,0027	Student t-test
Fig1.C-Arginase activity	Con vs Oleate	0,0029	Student t-test
Fig2.B Basal OCR	Con vs Oleate	<0.0001	Tukey's multiple comparisons test
Fig2.B Basal OCR	Oleate vs Stearate	<0.0001	Tukey's multiple comparisons test
Fig2.B Basal ECAR	Oleate vs Stearate	0,0012	Tukey's multiple comparisons test
Fig2.B Spare respiratory capacity	Con vs Oleate	0,0051	Tukey's multiple comparisons test
Fig2.B Spare respiratory capacity	Oleate vs Stearate	<0.0001	Tukey's multiple comparisons test
Fig2.B Proton leak	Con vs Oleate	<0.0001	Tukey's multiple comparisons test
Fig2.B Proton leak	Oleate vs Stearate	<0.0001	Tukey's multiple comparisons test
Fig2.B ATP production	Con vs Oleate	0,0001	Tukey's multiple comparisons test
Fig2.B ATP production	Oleate vs Stearate	0,0001	Tukey's multiple comparisons test
Fig2.B Maximum respiration	Con vs Oleate	<0.0001	Tukey's multiple comparisons test
Fig2.B Maximum respiration	Oleate vs Stearate	<0.0001	Tukey's multiple comparisons test
Fig2.C Basal OCR	Con vs Oleate	<0.0001	Tukey's multiple comparisons test
Fig2.C Spare respiratiory capacity	Con vs Con+ETO	0,0222	Tukey's multiple comparisons test
Fig2.C Spare respiratiory capacity	Con vs Oleate	<0.0001	Tukey's multiple comparisons test
Fig2.C Spare respiratiory capacity	Oleate vs Oleate+ETO	0,0034	Tukey's multiple comparisons test
Fig2.C Proton leak	Con vs Oleate	0,0043	Tukey's multiple comparisons test
Fig2.C ATP production	Con vs Oleate	<0.0001	Tukey's multiple comparisons test
Fig2.C ATP production	Oleate vs Oleate+ETO	0,0128	Tukey's multiple comparisons test
Fig2.C Maximum respiration	Con vs Oleate	0,0157	Tukey's multiple comparisons test
Fig2.C Maximum respiration	Con vs Con+ETO	<0.0001	Tukey's multiple comparisons test
Fig2.C Maximum respiration	Oleate vs Oleate+ETO	0,0031	Tukey's multiple comparisons test
Fig2.F %CD206	Con vs Oleate	0,0004	Tukey's multiple comparisons test
Fig2.F %CD206	Oleate vs Oleate+ETO	<0.0001	Tukey's multiple comparisons test
Fig2.F %CD206	Stearate vs Stearate+ETO	0,0375	Tukey's multiple comparisons test
Fig2.F MFI CD38	Con vs Oleate	<0.0001	Tukey's multiple comparisons test
Fig2.F MFI CD38	Oleate vs Oleate+ETO	<0.0001	Tukey's multiple comparisons test
Fig2.F MFI CD38	Oleate vs Stearate	<0.0001	Tukey's multiple comparisons test
Fig2.F MFI CD73	Con vs Oleate	<0.0001	Tukey's multiple comparisons test
Fig2.F MFI CD73	Oleate vs Oleate+ETO	<0.0001	Tukey's multiple comparisons test
Fig2.F MFI CD73	Oleate vs Stearate	<0.0001	Tukey's multiple comparisons test
Fig2.H %Divided	Oleate vs Oleate+ETO	0,0238	Tukey's multiple comparisons test
Fig2.I % Nitric Oxide	Oleate vs Oleate+ETO	<0.0001	Tukey's multiple comparisons test
Fig2.I % Nitric Oxide	Stearate vs Stearate+ETO	<0.0001	Tukey's multiple comparisons test

Fig3.B Basal OCR	Oleate vs Oleate+iDGAT	0,0005	Tukey's multiple comparisons test
Fig3.B Basal OCR	Oleate vs Oleate+MJN	<0.0001	Tukey's multiple comparisons test
Fig3.B ATP production	Oleate vs Oleate+iDGAT	0,0009	Tukey's multiple comparisons test
Fig3.B ATP production	Oleate vs Oleate+Atg	0,0049	Tukey's multiple comparisons test
Fig3.B ATP production	Oleate vs Oleate+MJN	<0.0001	Tukey's multiple comparisons test
Fig.3C CD206%	Con vs Con+MJN110	0,0192	Tukey's multiple comparisons test
Fig.3C CD206%	Oleate vs Oleate+iDGAT	<0.0001	Tukey's multiple comparisons test
Fig.3C CD206%	Oleate vs Oleate+Atg	0,0006	Tukey's multiple comparisons test
Fig.3C CD206%	Oleate vs Oleate+MJN	<0.0001	Tukey's multiple comparisons test
Fig.3D % Divided	Con vs Oleate	<0.0001	Tukey's multiple comparisons test
Fig.3D % Divided	Oleate vs Oleate+MJN	0,012	Tukey's multiple comparisons test
Fig.3D Proliferation Index	Con vs Oleate	<0.0001	Tukey's multiple comparisons test
Fig.3D Proliferation Index	Oleate vs Oleate+MJN	0,0008	Tukey's multiple comparisons test
Fig.3D Proliferation Index	Oleate vs Oleate+Atg	0,0007	Tukey's multiple comparisons test
Fig.3E	Oleate vs Oleate+Atg	<0.0001	Tukey's multiple comparisons test
Fig.3F M:T=1:30	Con vs Oleate	<0.0001	Tukey's multiple comparisons test
Fig.3F M:T=1:30	Oleate vs Oleate+MJN	<0.0001	Tukey's multiple comparisons test
Fig.3F M:T=1:30	Oleate vs Oleate+Atg	<0.0001	Tukey's multiple comparisons test
Fig.3F M:T=1:60	Con vs Oleate	<0.0001	Tukey's multiple comparisons test
Fig.3F M:T=1:60	Oleate vs Oleate+MJN	<0.0001	Tukey's multiple comparisons test
Fig.3F M:T=1:60	Oleate vs Oleate+Atg	<0.0001	Tukey's multiple comparisons test
Fig.4A	Oleate vs Oleate+Rapa	0,0128	Tukey's multiple comparisons test
Fig.4B Proliferation Index	Oleate vs Oleate+Rapa	0,0097	Tukey's multiple comparisons test
Fig.4B Nitric Oxide M:T=1：30	Con vs Con+Rapa	0,0477	Tukey's multiple comparisons test
Fig.4B Nitric Oxide M:T=1：30	Con vs Oleate	0,0058	Tukey's multiple comparisons test
Fig.4B Nitric Oxide M:T=1：30	Oleate vs Oleate+Rapa	0,0017	Tukey's multiple comparisons test
Fig.4B Nitric Oxide M:T=1：60	Con vs Oleate	0,0041	Tukey's multiple comparisons test
Fig.4B Nitric Oxide M:T=1：60	Oleate vs Oleate+Rapa	0,0011	Tukey's multiple comparisons test
Fig.4B % of divided	Oleate vs Oleate+Rapa	0,0251	Tukey's multiple comparisons test
Fig.4C Basal OCR	Con vs Oleate	0,045	Tukey's multiple comparisons test
Fig.4C Basal OCR	Oleate vs Oleate+Rapa	<0.0001	Tukey's multiple comparisons test
Fig.4C Spare respiratiory capacity	Con vs Oleate	0,0006	Tukey's multiple comparisons test
Fig.4C Spare respiratiory capacity	Con vs Con+Rapa	<0.0001	Tukey's multiple comparisons test
Fig.4C Spare respiratiory capacity	Oleate vs Oleate+Rapa	<0.0001	Tukey's multiple comparisons test
Fig.4C Maximum Repsipration	Con vs Con+Rapa	0,0014	Tukey's multiple comparisons test
Fig.4C Maximum Repsipration	Oleate vs Oleate+Rapa	<0.0001	Tukey's multiple comparisons test
Fig.4C ATP production	Con vs Oleate	0,0063	Tukey's multiple comparisons test
Fig.4C ATP production	Oleate vs Oleate+Rapa	<0.0001	Tukey's multiple comparisons test

Fig.4D Gr1-CD11b+	day6 Con vs Oleate	0,0074	Bonferroni's multiple comparisons test
Fig.4D Gr1+CD11b+	day6 Con vs Oleate	0,0297	Bonferroni's multiple comparisons test
Fig.5 A MCA205	Con vs Oleate	0,0266	Student t-test
Fig.5 A CT26	Con vs Oleate	<0.0001	Student t-test
Fig.5 B Day12	Con vs Oleate	0,0453	Bonferroni's multiple comparisons test
Fig.5 B Day14	Con vs Oleate	0,0028	Bonferroni's multiple comparisons test
Fig.5 D Overall	Liposome vs Liposome +iDGAT	0,0405	Two-way ANOVA
Fig.5 D Day 28	Liposome vs Liposome +iDGAT	0,0004	Bonferroni's multiple comparisons test
Fig.5 E	Tumor Lipo vs Tumor Lipo+iDGAT	0,0039	Tukey's multiple comparisons test
Fig.5 F	PBS vs. Liposome+iDGAT	<0.0001	Tukey's multiple comparisons test
Fig.5 F	Liposome+iDGAT vs. Liposome	<0.0001	Tukey's multiple comparisons test
Fig.6A CD206	Con vs Oleate	<0.0001	Tukey's multiple comparisons test
Fig.6A CD206	Oleate vs Stearate	<0.0001	Tukey's multiple comparisons test
Fig.6A CD206	Con vs Stearate	0,0004	Tukey's multiple comparisons test
Fig.6A CD204	Con vs Oleate	0,0171	Tukey's multiple comparisons test
Fig.6A CD204	Oleate vs Stearate	0,0181	Tukey's multiple comparisons test
Fig.6A CD38	Con vs Oleate	0,0007	Tukey's multiple comparisons test
Fig.6A CD38	Oleate vs Stearate	<0.0001	Tukey's multiple comparisons test
Fig.6D	Con vs Oleate	0..0036	Tukey's multiple comparisons test
Fig.6D	Oleate vs Oleate+iDGAT	0,03	Tukey's multiple comparisons test
Fig.6E	Control vs Tumor	0,0374	Student t-test
Fig. S1B	Neg_Con vs Oleate	0,0072	Two-way ANOVA
Fig. S1D ratio of CD4+/CD8+	Tumor neg-Con vs Tumor Oleate	<0.0001	Tukey's multiple comparisons test
Fig. S1D ratio of CD4+/CD8+	Tumor Con vs Tumor Oleate	0,0005	Tukey's multiple comparisons test
Fig. S1D % of CD4	Tumor neg-Con vs Tumor Oleate	<0.0001	Tukey's multiple comparisons test
Fig.S2A % Divided	Oleate vs Oleate+Cele	0,0014	Two-way ANOVA
Fig.S2A Nitric Oxide	Oleate vs Oleate+Cele	0,0481	Two-way ANOVA
Fig.S2B	Oleate vs Oleate+Cele	0,0411	Tukey's multiple comparisons test
Fig. S3A Basal OCR	Con vs Con+CAY	0,0004	Tukey's multiple comparisons test
Fig. S3A Basal OCR	Con vs Oleate	<0.0001	Tukey's multiple comparisons test
Fig.S3A Spare respiratiory capacity	Con vs Con+CAY	0,0029	Tukey's multiple comparisons test
Fig.S3A Proton Leak	Con vs Con+CAY	<0.0001	Tukey's multiple comparisons test
Fig.S3A ATP Production	Con vs Con+CAY	<0.0001	Tukey's multiple comparisons test
Fig.S3A Maximal Respiration	Con vs Con+CAY	0,0042	Tukey's multiple comparisons test
Fig.S5B Proportion	Con vs Con+CAY	0,0001	Tukey's multiple comparisons test
Fig.S5B Proportion	Con vs Oleate	0,0085	Tukey's multiple comparisons test
Fig.S5B Relative cell number	Con vs Con+CAY	<0.0001	Tukey's multiple comparisons test
Fig.S5B Relative cell number	Con vs Oleate	0,0055	Tukey's multiple comparisons test
Fig. S4B Lipo-	Con vs Oleate	<0.0001	Tukey's multiple comparisons test

Fig. S4B Lipo-	Oleate vs Oleate+iDGAT	<0.0001	Tukey's multiple comparisons test
Fig. S4B Lipo+	Con vs Oleate	0,0003	Tukey's multiple comparisons test
Fig. S4B Lipo+	Oleate vs Oleate+iDGAT	<0.0001	Tukey's multiple comparisons test
Fig. S4B Mouse spleen CD11b+	Oleate vs Oleate+iDGAT	0,0022	Tukey's multiple comparisons test
Fig. S4B Human CD14+	Oleate vs Oleate+iDGAT	0,0185	Tukey's multiple comparisons test
Fig. S5A % CD206+	Con vs Oleate	0,0014	Student t-test
Fig. S5A % CD206+ in CD45.1	Con vs Oleate	0,0014	Student t-test
Fig. S5A Absolute number	Con vs Oleate	0,0036	Student t-test
Fig. S5B % CD206+	Con vs Oleate	0,0005	Student t-test
Fig. S5B Absolute number	Con vs Oleate	0,0026	Student t-test

Table S3. Supplementary Material and Methods: Exact p-values and statistical tests.
